
JOURNAL INFORMATION
==============================
NLM Title Abbreviation: Wirtschaftsdienst
Journal ID: Wirtschaftsdienst
NLM Journal ID: 101086612

Wirtschaftsdienst (Hamburg, Germany : 1949)

ISSN: 0043-6275

ARTICLE INFORMATION
==============================
PMCID: PMC7237608
PMID: 32454539
DOI: 10.1007/s10273-020-2646-y
Article ID: 2646
Article version: 1
Subjects: Zeitgespräch

WTO-Reform: Formale Regelgleichheit reicht nicht aus, alle Mitgliedsländer brauchen gleiche wirtschaftliche Chancen

Klein Martin martin.klein@wiwi.uni-halle.de

grid.9018.0 0000 0001 0679 2801 International Economics, Martin-Luther-Universität Halle-Wittenberg, Große Steinstr. 73, 6108 Halle (Saale), Deutschland
Electronic publication date: 2020 May 20
Print publication date: 2020
Volume: 100
Issue: 5
First page: 315
Last page: 320
Copyright: © Der/die Autor(en) 2020
License: Open Access Dieser Artikel wird unter der Creative Commons Namensnennung 4.0 International Lizenz veröffentlicht, welche die Nutzung, Vervielfältigung, Bearbeitung, Verbreitung und Wiedergabe in jeglichem Medium und Format erlaubt, sofern Sie den/die ursprünglichen Autor(en) und die Quelle ordnungsgemäß nennen, einen Link zur Creative Commons Lizenz beifügen und angeben, ob Änderungen vorgenommen wurden.
Die in diesem Artikel enthaltenen Bilder und sonstiges Drittmaterial unterliegen ebenfalls der genannten Creative Commons Lizenz, sofern sich aus der Abbildungslegende nichts anderes ergibt. Sofern das betreffende Material nicht unter der genannten Creative Commons Lizenz steht und die betreffende Handlung nicht nach gesetzlichen Vorschriften erlaubt ist, ist für die oben aufgeführten Weiterverwendungen des Materials die Einwilligung des jeweiligen Rechteinhabers einzuholen.
Weitere Details zur Lizenz entnehmen Sie bitte der Lizenzinformation auf http://creativecommons.org/licenses/by/4.0/deed.de.
License URL: https://creativecommons.org/licenses/by/4.0/

issue-copyright-statement: © The Author(s) 2020

==============================

Die Entwicklung der multilateralen Ordnung von der Nachkriegszeit bis heute wird gerne als Erfolgsgeschichte erzählt. Nach zwei Weltkriegen war es gelungen, eine weltwirtschaftliche Ordnung zu schaffen, die den teilnehmenden Ländern freien Handel, Wohlstand und Frieden sicherte. Die Welthandelsorganisation (WTO) und ihre Vorläuferorganisation, das Allgemeine Zoll- und Handelsabkommen GATT (General Agreement on Tariffs and Trade), waren ein integraler Teil dieser Erfolgsgeschichte. Im Verbund mit dem Internationalen Währungsfonds (IWF), der Weltbank und vielen anderen größeren und kleineren internationalen Organisationen bildeten sie das institutionelle Exoskelett der Weltwirtschaft.

Anders als der Beitritt zu den Bretton-Woods-Organisationen IWF und Weltbank war und ist der Beitritt zu GATT/WTO eher mühsam. Beitrittskandidaten müssen mit allen interessierten Mitgliedsländern Handelskonzessionen vereinbaren, um am Ende grünes Licht für den Beitritt zu erhalten. Bei großen Ländern (vor allem bei Russland und China) waren die Beitrittsverfahren politisch überfrachtet. Trotz dieser Schwierigkeiten ist die Mitgliederzahl des GATT bzw. der WTO im Laufe der Jahre von den 23 Vertragsparteien des GATT auf nunmehr 164 Länder angestiegen.

Die Wachstumskurve der Mitgliederzahl von GATT und WTO zeichnet eine Geschichte der Globalisierung nach (vgl. Abbildung 1). Der erste Schub der Globalisierung nach dem Krieg war die Gründung des GATT, die parallel zur Gründung der Vereinten Nationen, des IWF und der Weltbank stattfand und damit in einer Art „Urknall“ die Basis der Nachkriegsordnung legte. Sie ermöglichte auch der Bundesrepublik erste Schritte zur Reintegration nicht nur in die Weltwirtschaft, sondern überhaupt in die internationale Politik. Der deutsche Beitritt zur WTO 1951 erfolgte ein Jahr vor dem Beitritt zum IWF und der Weltbank und 22 Jahre vor dem Beitritt zu den Vereinten Nationen. Der GATT-Beitritt Japans, das sich in einer ähnlichen Lage wie Deutschland befand, brachte 1955 die erste Phase der Nachkriegsglobalisierung zum Abschluss (Rode, 2006).

Die zweite Globalisierungsphase wurde durch die Dekolonialisierung Afrikas vorangetrieben. Den Anfang machte Ghana, das 1957 Mitglied des GATT wurde, kaum sechs Monate, nachdem es seine Unabhängigkeit von Großbritannien erlangt hatte. In schneller Folge traten die neuen Länder Afrikas dem GATT bei, bis diese Phase 1966 mit dem Beitritt Ruandas zu Ende ging.

Erst 1986 mit dem Beginn der Uruguay-Runde in Punta del Este nahm die Globalisierung wieder Fahrt auf. Nachdem unter den US-Präsidenten Bush und Clinton entscheidende Durchbrüche bei den Verhandlungen erzielt worden waren, gewann das GATT in schneller Folge neue Länder hinzu, die sich als Mitglieder einen Einfluss auf das Verhandlungsergebnis und den Zugang zur WTO sichern wollten. Die Gründung der WTO, die ihre Arbeit Anfang 1995 aufnahm, schloss diese Phase ab.

In den folgenden Jahren stieg die Mitgliederzahl der WTO in eher gemächlichem Rhythmus an, wobei allerdings 2001 der Beitritt Chinas zur WTO einen letzten wichtigen Meilenstein der Globalisierung bildete. Wie der Bundesverband der Deutschen Industrie (2019) schreibt, wurden mit Chinas Beitritt die Hoffnungen verbunden, dass die Einbindung in die multilaterale Organisation als Katalysator für Strukturreformen wirken und freies unternehmerisches Handeln fördern würde. Diese Hoffnungen wurden weitestgehend enttäuscht.

Nach fast 75 Jahren als Treiber der Globalisierung steht die WTO heute vor der größten Herausforderung ihrer Geschichte. Sie steht in Gefahr, für den Welthandel irrelevant zu werden. Eine Ursache ist die Boykotthaltung, die die USA unter Präsident Trump gegenüber der WTO und insbesondere gegenüber ihrem Streitschlichtungsverfah-

Abbildung 1
**GATT-/WTO-Beitrittsgeschichte**

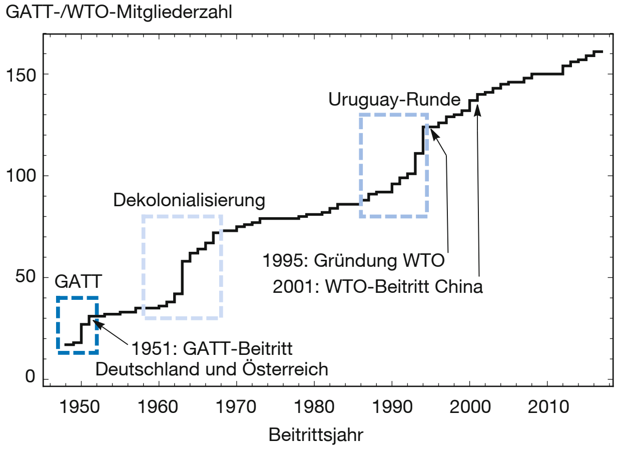

Quelle: WTO.

Abbildung 2
**Netzwerk der WTO-Streitschlichtung**

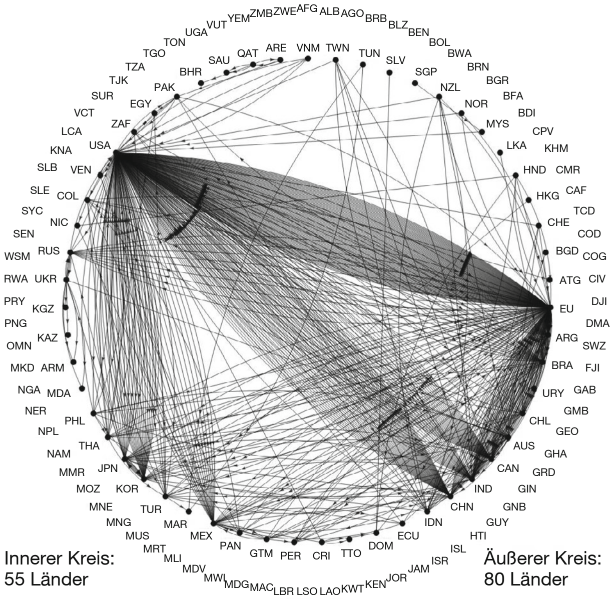

Die Mitgliedsländer der WTO werden durch ihre ISO-Kürzel dargestellt.

Quelle: WTO und eigene Berechnungen.

ren einnehmen. Eine weitere Ursache ist der dramatische Einbruch des Welthandels durch die COVID-19-Pandemie. Die WTO (2020) selbst hat unlängst vor einem Einbruch des Welthandels zwischen 13 % und 32 % gewarnt. Schon ist die Rede von der Renationalisierung der Wertschöpfungsketten (Reshoring), von Deglobalisierung und einem Wiedererstarken des Protektionismus. Damit treibt die Pandemie genau jene unheilvollen Tendenzen an, zu deren Eindämmung GATT und WTO einst geschaffen wurden.

## Die Misere der WTO-Streitschlichtung

Die Boykottpolitik der Trump-Regierung gegenüber der WTO-Streitschlichtung begann schon 2017 durch Blockierung der Nominierung nachrückender Mitglieder für das Berufungsgremium (Appellate Body) der Streitschlichtung. Im Dezember 2019 war das Berufungsgremium dadurch auf ein Mitglied geschrumpft und damit unter die Mindestzahl von drei Mitgliedern, die für eine Berufungsentscheidung erforderlich ist. Nach eigenem Bekunden will die Trump-Regierung damit eine tiefgreifende Reform der WTO erzwingen.

Die Europäische Union war federführend bei der Schaffung einer Notlösung, die darauf abzielt, eine Art „Ersatz-Berufungsverfahren" für teilnahmewillige WTO-Mitglieder zu schaffen. Das entsprechende „Multi-party interim appeal arbitration arrangement" (MPIA) liegt seit Ende März 2020 vor. Außer der EU nehmen 16 weitere WTO-Mitglieder daran teil, darunter auch China. Es ist allerdings fraglich, ob das MPIA eine große Wirkung entfalten wird. Die Trump-Regierung geht inzwischen so weit, dass sie sich an keine Beschlüsse der WTO-Streitschlichtung mehr gebunden fühlt, weil – so ihr Argument – das Schiedsverfahren nicht mehr WTO-rechtskonform sei.[1]

In dieser Lage ist ein einfaches „weiter so" nicht möglich. Die Streitschlichtung der WTO wird an einer Reform nicht vorbeikommen. Und dabei werden natürlich auch andere Probleme ins Blickfeld geraten. Was auffällt, ist vor allem die extreme Ungleichheit bei der Nutzung der Streitschlichtung. Abbildung 2 zeigt die Streitschlichtung der WTO als ein Netzwerk von Klägern und Beklagten (claimants/respondents). Alle Mitgliedsländer der EU (die nach den Regeln der WTO auch einzeln Ziel von Handelsstreitigkeiten werden können) wurden zur besseren Übersichtlichkeit der EU zugeschlagen. Ein Pfeil z. B. von den USA zur EU repräsentiert einen Streitfall, bei dem die USA der Europäischen Union oder einem ihrer Mitgliedsländer eine Verletzung der Verpflichtungen, die diese durch ihren Beitritt zur WTO eingegangen sind, vorwirft. Abbildung 2 verdeutlicht eine extrem ungleiche Verteilung der Streitfälle. USA und EU nehmen die Streitschlichtung mit Abstand am häufigsten wahr, und sie streiten sich vor allem miteinander. Weit dahinter kommen China, Indien und ein paar andere Länder. Fast die Hälfte der WTO-Mitglieder

1 Das jüngste Beispiel betrifft einen Streitfall mit Kläger Kanada, in dem die WTO entschieden hat, dass die USA ihre Zölle auf bestimmte Papiersorten abschaffen müssen. Vgl. dazu Baschuk (2020).

waren noch nie in die Streitschlichtung involviert. Sie schauen der Streitschlichtung, wie in Abbildung 2 veranschaulicht, gleichsam unbeteiligt zu.

Woher kommt diese extreme Ungleichheit bei der Inanspruchnahme der Streitschlichtung? Sollte man nicht meinen, dass gerade die kleineren und ärmeren Länder zu den größten Nutznießern der WTO zählen, wie Felbermayr (2019) vor kurzem meinte? Tatsächlich kann das vermeintlich rechtsgleiche Regelsystem der Streitschlichtung sehr ungleiche Wirkungen haben. Von Rechtswissenschaftlern wird schon lange darauf hingewiesen, dass die Teilnahme an der Streitschlichtung einen hohen Kostenaufwand und erheblichen juristischen Sachverstand in diesem hochspezialisierten Rechtsgebiet erfordert. Reiche Länder können sich bei Handelsstreitigkeiten von spezialisierten Kanzleien beraten lassen, die Kosten dafür stellen für sie keinen Hinderungsgrund dar. In den ärmeren Mitgliedsländern der WTO können diese Faktoren jedoch prohibitiv sein. Die Debatte zu diesen Problemen ist nicht neu (z. B. Shaeffer, 2005 und aus afrikanischer Sicht Mugota, 2017).

Wichtiger sind dabei aber die ökonomischen Aspekte. Streitfälle werden nicht abschließend durch die WTO entschieden, vielmehr spielt diese nur eine subsidiäre Rolle, indem ihr Schiedsgericht einer der streitenden Parteien das Recht auf Vergeltungsmaßnahmen in Form von Strafzöllen einräumt. Länder, denen vom Schiedsgericht Recht zugesprochen wird, dürfen ihren Kontrahenten den Zugang zu ihrem eigenen Markt verwehren, indem sie auf deren Produkte Strafzölle erheben. Die wenigsten Streitfälle enden tatsächlich mit der Erhebung von Strafzöllen, meist einigt man sich im Vorfeld oder im Verlauf des Verfahrens auf einen Kompromiss. Spieltheoretiker würden das als Verhandlungsspiel bezeichnen, dessen Ausgang von der Verhandlungsmacht der Kontrahenten abhängt. Das „Vorführen der Folterinstrumente" gehört zum Verhandlungsprozess. Länder bzw. Regionen mit großen Absatzmärkten sind dabei klar im Vorteil. Wenn die USA oder die Europäische Union Strafzölle erheben, dann fügen sie den betroffenen Handelspartnern erhebliche wirtschaftliche Schäden zu. Deshalb ist allein schon die Androhung von Strafzöllen ein überaus wirksames Druckinstrument, um im Rahmen einer Streitschlichtung Forderungen durchzusetzen. Wenn umgekehrt ein armes Entwicklungsland den USA oder der EU androht, durch Strafzölle den Zugang zu seinem heimischen Absatzmarkt zu entziehen, so wird sich der Schrecken darüber in Grenzen halten. Die Größenunterschiede beim Einkommen pro Kopf und auch bei der Bevölkerung sind dermaßen exorbitant, dass die formale Rechtsgleichheit, die der Streitschlichtung zugrunde liegt, im Ergebnis zunichte gemacht wird. Bei extremer wirtschaftlicher Ungleichheit führt formale Rechtsgleichheit in der Realität zu Rechtsungleichheit. Gerade für die kleineren und ärmeren

Abbildung 3
**Südkorea, Katar, China – BIP pro Kopf und GATT-/WTO-Beitritt**

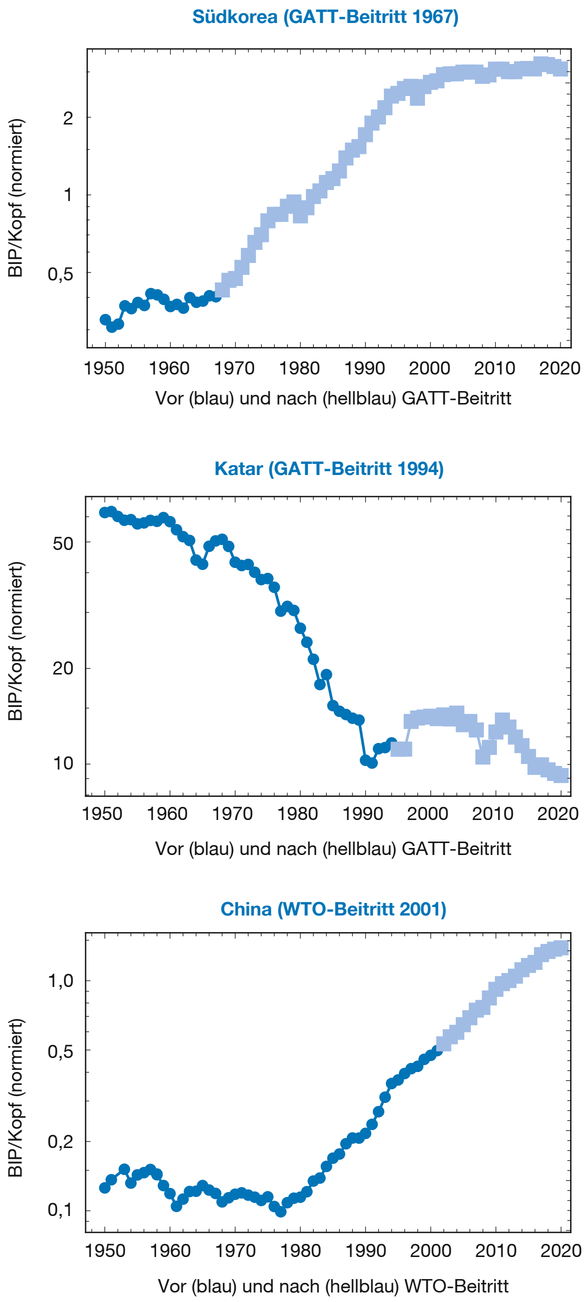

Anmerkung: Das Bruttoinlandsprodukt (BIP) wird durch den Vergleich mit dem Median des BIP aller Länder im jeweiligen Jahr normiert. Der Wert von 1 auf der vertikalen Achse bedeutet, dass 50 % aller Länder ein höheres, und ebenfalls 50 % ein geringes BIP aufwiesen.

Quelle: Die BIP-Daten stammen in der Mehrheit aus dem World Economic Outlook des IWF. In Jahren, wo in dieser Quelle für bestimmte Länder keine Daten zur Verfügung standen, wurden Daten der Penn World Tables (PWT) verwendet. Wo auch keine PWT-Daten vorhanden waren, wurde Daten aus dieser Quelle verwendet: James et al. (2012).

Abbildung 4

**Asien und Afrika – relative Einkommensentwicklung nach GATT-/WTO-Beitritt**

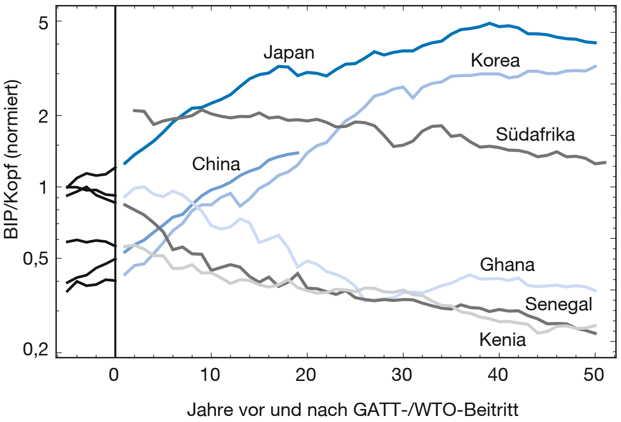

Die horizontale Achse zeigt nicht Kalenderjahre, sondern die Jahre vor und nach dem Beitritt des jeweiligen Landes. Das Jahr des Beitritts wird mit 0 bezeichnet. In Südafrika gibt es nur Daten nach dem Beitritt, weil das Land dem GATT schon 1948 beitrat. Es gehört zu den ursprünglichen Vertragsparteien (contracting parties) des GATT. In China stehen seit dem WTO-Beitritt nur Daten für 20 Jahre zur Verfügung.

Quelle: Die BIP-Daten stammen in der Mehrheit aus dem World Economic Outlook des IWF. In Jahren, wo in dieser Quelle für bestimmte Länder keine Daten zur Verfügung standen, wurden Daten der Penn World Tables (PWT) verwendet. Wo auch keine PWT-Daten vorhanden waren, wurde Daten aus dieser Quelle verwendet: James et al. (2012).

Abbildung 5

**USA, Europa und Südamerika – relative Einkommensentwicklung nach GATT-/WTO-Beitritt**

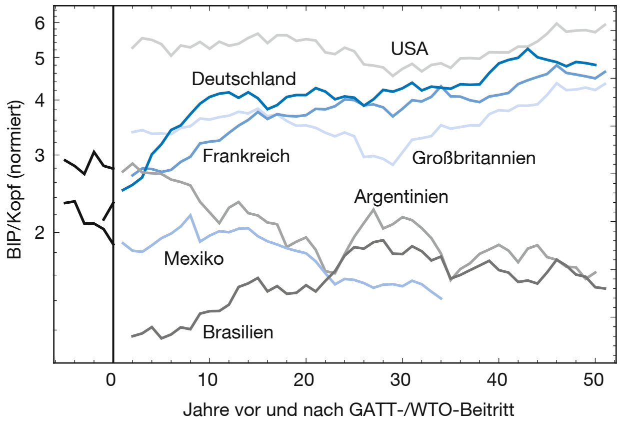

Quelle: Die BIP-Daten stammen in der Mehrheit aus dem World Economic Outlook des IWF. In Jahren, wo in dieser Quelle für bestimmte Länder keine Daten zur Verfügung standen, wurden Daten der Penn World Tables (PWT) verwendet. Wo auch keine PWT-Daten vorhanden waren, wurde Daten aus dieser Quelle verwendet: James et al. (2012).

Mitgliedsländer die WTO bleibt Rechtsgleichheit bei der Schlichtung von Handelsstreitigkeiten eine Fiktion.

## Ungleichheit in der WTO

Wie steht es mit der wirtschaftlichen Ungleichheit in der WTO? Haben GATT und WTO im Laufe ihrer fast 75-jährigen Geschichte ihren Mitgliedsländern den wirtschaftlichen Aufstieg erlaubt, oder vertiefen sich die Gräben zwischen arm und reich? Antworten auf diese Fragen sind nicht einfach, und sie müssen auf die jeweils besonderen Umstände in den Ländern eingehen. Zuerst ist festzuhalten, dass die WTO unter ihren Mitgliedsländern zahlreiche Erfolgsgeschichten aufzuweisen hat. Abbildung 3 zeigt drei Beispiele: die Entwicklung des normierten realen Bruttoinlandsprodukts (BIP) pro Kopf in Südkorea, Katar und China vor und nach dem Beitritt zur WTO. Südkorea trat in dieser Vergleichsgruppe zuerst dem GATT bei. Die Daten zeigen eine dramatische Erfolgsgeschichte, die das Land von einem der ärmeren der Welt zu einem der reichsten aufsteigen ließen. Der Beitritt zum GATT war entscheidend für die Wirtschaftswende der Öffnung und Modernisierung, die den wirtschaftlichen Wandel antrieb. Unter völlig anderen Vorzeichen war auch in Katar der Beitritt zum GATT (im letzten Jahr vor Gründung der WTO) ein wichtiger Baustein in der Reformagenda, die auf eine Diversifizierung der vorher rein auf Rohöl ausgerichteten Wirtschaft abzielte. Das Land konnte dadurch seinen relativen Abstieg stoppen und seine Position auf hohem Niveau stabilisieren. China schließlich trat der WTO spät bei. Es befand sich schon seit den 1980er Jahren auf Wachstumskurs. Der Beitritt zur WTO diente dem schnell wachsenden Land dazu, den Zugang vor allem zu den Märkten der Industrieländer abzusichern und damit die Fortsetzung des exportorientierten Wachstums zu ermöglichen.

Drei unterschiedliche Länder, drei unterschiedliche Erfolgsgeschichten. Doch in dieser Metrik kann es nicht nur Gewinner geben. Abbildung 4 vergleicht in Form einer Event-Analyse die relative BIP-Entwicklung ab dem Jahr des Beitritts zu GATT/WTO in Asien und in Afrika. Abgesehen von Südafrika finden sich alle Länder im Jahr ihres Beitritts in der unteren Hälfte der weltweiten Einkommensverteilung. 50 Jahre danach haben sich die Länder klar getrennt. Die asiatischen Länder verzeichnen starke Zugewinne, die afrikanischen Länder starke Verluste. Für die einen wurde der Beitritt zum GATT eine Erfolgsgeschichte, für die anderen brachte er 50 Jahre Enttäuschungen.

Ein ähnliches Problem zeigt sich, wenn wir die relative Entwicklung Südamerikas betrachten (vgl. Abbildung 5). Wir bewegen uns hier in der oberen Hälfte der weltweiten

Einkommensverteilung (d. h. alle Länder liegen über dem Medianeinkommen), doch auch hier bildet sich in den 50 Jahren seit dem Beitritt zum GATT in zunehmendem Maße eine Lücke zu den ausgewählten europäischen Ländern und den USA heraus, die eindeutig zu den Gewinnern gehören. Besonders dramatisch ist dies bei Argentinien, das bei seinem GATT-Beitritt in einer besseren Position war als die Deutschland, 50 Jahre danach aber auf das Einkommensniveau eines Schwellenlands abgesunken ist.

Abbildung 6 zeigt, dass das Problem der wachsenden Ungleichheit unter dem GATT nicht nur einer willkürlichen Länderauswahl geschuldet ist. Die obere Abbildung 6 kontrastiert die Einkommensentwicklung der Länder in der ersten Beitrittswelle zum GATT mit der Einkommensentwicklung in der zweiten Beitrittswelle (untere Abbildung 6). In der ersten Gruppe befinden sich die wichtigsten Industrieländer der damaligen Zeit, in der zweiten Gruppe überwiegend die gerade unabhängig gewordenen Länder Afrikas. Im Verlauf von 50 Jahren nach dem Beitritt bewegt sich die Einkommensposition der ersten Gruppe nach rechts, d. h. sie werden relativ zum weltweiten Median noch reicher, während die Einkommensposition der zweiten Gruppe immer weiter absinkt. Im Laufe der Jahre klafft zunehmend eine Einkommenslücke zwischen den beiden Gruppen.

Abbildung 6
**GATT-Beitritt für afrikanische Länder wenig erfolgreich**

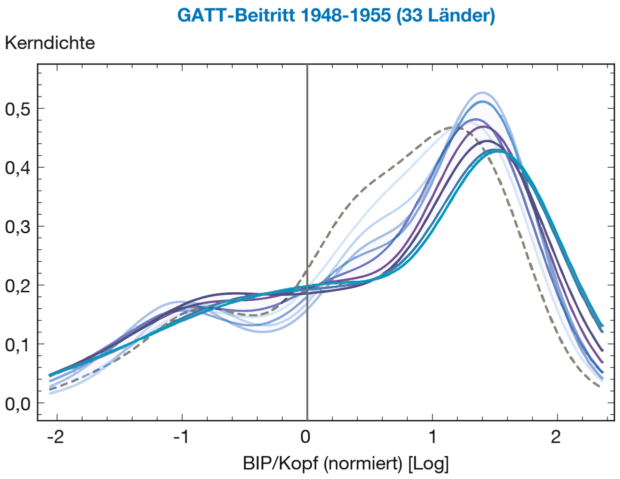

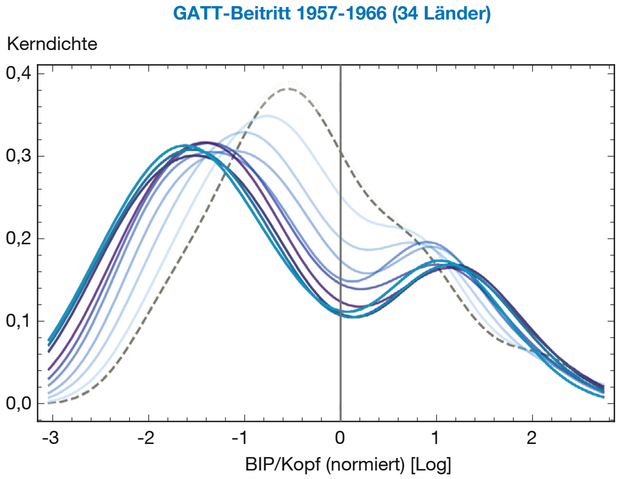

Dabei werden die 50 Jahre nach dem GATT-Beitritt in zehn Fünfjahresscheiben zerlegt und für jede von ihnen eine Häufigkeitsverteilung (Kerndichte) berechnet. Die erste Verteilung des BIP in der Periode des Beitrittsjahres wird durch die gestrichelte Linie wiedergegeben. Die Verteilungen der Folgeperioden sind durch unterschiedliche Farbgebung schrittweise davon abgesetzt. Das weltweite Medianeinkommen wird hier durch den Wert Null dargestellt.

Quelle: Die BIP-Daten stammen in der Mehrheit aus dem World Economic Outlook des IWF. In Jahren, wo in dieser Quelle für bestimmte Länder keine Daten zur Verfügung standen, wurden Daten der Penn World Tables (PWT) verwendet. Wo auch keine PWT-Daten vorhanden waren, wurde Daten aus dieser Quelle verwendet: James et al. (2012).

## Wie geht es mit der WTO weiter?

Fast 75 Jahre nach der Gründung des GATT steht die WTO vor der größten Herausforderung ihrer Geschichte. COVID-19-Pandemie, Einbruch des Welthandels, Renationalisierung der Wertschöpfungsketten und vor allem die Boykotthaltung der USA müssen nicht unbedingt zu einer Abschaffung der WTO führen, wohl aber zu ihrem Absinken in die Irrelevanz. Es ist unbestritten, dass die WTO reformbedürftig ist, ein einfaches „weiter so" ist nicht möglich.

Die Europäische Union versucht gegenzusteuern und hat mit anderen WTO-Mitgliedern eine Zwischenlösung zur Rettung der Streitschlichtung vereinbart. Doch außer der EU haben sich nur 16 Länder dazu bereitgefunden, 148 bleiben freiwillig außen vor, darunter z. B. auch Indien, das als Meinungsführer unter den Entwicklungs- und Schwellenländern in der WTO eine gewichtige Position einnimmt. Dies ist ein klares Zeichen, dass die Mehrheit der WTO-Mitglieder nicht bereit ist, politisches Kapital für eine Rettung der Streitschlichtung zu investieren. Sie mögen nicht die Ziele und Methoden der USA unter Präsident Trump billigen, doch auch sie sind mit dem Status quo der WTO unzufrieden.

Erinnern wir uns daran, dass sich die WTO immer noch in der 2001 begonnenen Verhandlungsrunde von Doha befindet, die auch als Doha-Development-Agenda bezeichnet wird, weil ihr Ziel eine grundlegende Verbesserung der Lage der Entwicklungsländer im Welthandel ist. Weil sich die Hauptkontrahenten nicht einigen konnten, konnte die Doha-Runde noch nicht abgeschlossen werden. Es muss auch der EU und ihren Mitgliedsländern klar sein: Solange die Industrieländer nicht bereit sind, auf die Entwicklungs- und Schwellenländer zuzugehen, wird es keine WTO-Reform geben.

Der Autor gibt seine persönliche Meinung wieder.

Prof. Dr. Martin Klein ist Professor für International Economics an der Martin-Luther-Universität Halle-Wittenberg.


Literatur

Rode, R. (2006), Kluge Handelsmacht. Gezähmte Liberalisierung als Gover-nanceleistung im Welthandelsregime GATT/WTO, LIT Verlag.
Bundesverband der Deutschen Industrie (BDI) (2019), China in der Welthandelsorganisation, https://bdi.eu/artikel/news/china-in-der-wto/ (27. April 2020).
World Trade Organization (WTO) (2020), Trade set to plunge as COVID-19 pandemic upends global economy, https://www.wto.org/english/news_e/pres20_e/pr855_e.htm (27. April 2020).
Baschuk, B. (2020), U.S. says WTO’s appellate body is invalid, balks at compliance, Bloomberg, 22. April, https://www.bloomberg.com/news/articles/2020-04-22/u-s-says-wto-s-appellate-body-is-inva-lid-balks-at-compliance (27. April 2020).
Felbermayr, G. (2019), 25 Jahre WTO — Ursachen des Zerfalls und Reformvorschläge für die Zukunft, https://www.ifw-kiel.de/de/publika-tionen/kiel-focus/2019/25-jahre-wto-ursachen-des-zerfalls-und-re-formvorschlaege-fuer-die-zukunft-0/ (27. April 2020).
Shaeffer, G. (2005), Developing Country Use of the WTO Dispute Settlement System: Why it Matters, the Barriers Posed, and its Impact on Bargaining.
Mugota, Z. V. (2017), A Critical Analysis if The World Trade Organization’s dispute settlement system is equally available to all member states and creates a fair and level playing field, https://zimlii.org/zw/journal/2016-zelj-01/%5Bnode%3Afield_jpubdate%3Acustom%3AY/critical-analysis-world-trade-organization%E2%80%99s (27. April 2020).
James S L Gubbins P Murray C J L Gakidou E Developing a comprehensive time series of GDP per capita for 210 countries from 1950 to 2015 Population Health Metrics 2012 10 1 12 10.1186/1478-7954-10-12 22846561 PMC3487911
World Trade Organization (WTO) (o. D.), The Doha Round, https://www.wto.org/english/tratop_e/dda_e/dda_e.htm (27. April 2020).
